# With or without internal limiting membrane peeling during idiopathic epiretinal membrane surgery: A meta-analysis

**DOI:** 10.1371/journal.pone.0245459

**Published:** 2021-01-19

**Authors:** Qinying Huang, Jinying Li

**Affiliations:** 1 Shantou University Medical College, Shantou, Guangdong, China; 2 Department of Ophthalmology, Peking University Shenzhen Hospital, Shenzhen, Guangdong, China; Massachusetts Eye & Ear Infirmary, Harvard Medical School, UNITED STATES

## Abstract

**Background:**

Although previously published meta-analyses have compared the surgical effects between the methods of Idiopathic epiretinal membrane (iERM) removal with or without ILM peeling, they did not reach an agreement.

**Purpose:**

We aimed to provide more evidence for the treatment of iERM and whether additional ILM peeling was better or not by analyzing more updated studies and randomized control trials (RCTs).

**Method:**

The search was conducted in Pubmed, Embase, Cochrane Library, Web of Science and Open Grey without language limitation and the studies included were from inception to December 2019. All studies of iERM with or without ILM peeling showed at least one of outcomes, such as best-corrected visual acuity (BCVA), central macular thickness (CMT) and recurrence of ERM. The pooled results between above groups were showed by the mean differences (MDs) and risk ratios (RR) with corresponding 95% confidence intervals (CIs).

**Result:**

In total, 1645 eyes of five randomized controlled trials (RCTs) and fifteen retrospective studies were included. The short-term (<12 months) BCVA improvement in both groups showed no significant difference (MD = -0.01; 95% CI = -0.02 to 0.01; P = 0.36). However, the BCVA improvement was significantly better in ILM peeling eyes than in those without ILM peeling when considering the risk bias (MD = -0.04; 95% CI = -0.07 to -0.01; P = 0.008). The short-term (<12 months) CMT had a higher reduction in non ILM peeling group (MD = -9.02; 95% CI = -12.51 to -5.54; P < 0.00001) and the recurrence of ERM in ILM peeling group was lower (P < 0.00001). The long-term (≥12months) BCVA improvement ((MD = -0.00; 95% CI = -0.03 to 0.03; P = 0.97) and reduction of long-term (≥12months) CMT (MD = -1.14; 95% CI = -7.14 to -4.86; P = 0.71) were similar in both groups.

**Conclusion:**

By considering the risk of bias, we should determine whether ILM peeling is beneficial for short-term changes in BCVA in patients with iERM. Nevertheless, further studies are needed to confirm this. iERM removal without ILM peeling can improve the short-term decrease in CMT and ILM peeling decreases the recurrence of ERM, but the long-term changes in BCVA and CMT are similar with or without ILM peeling. There is a need for a true large scale randomized trial that will also include microperimetry and other functional measures.

## 1. Background

Idiopathic epiretinal membrane (iERM), a sheet of fibrotic tissue found at the vitreoretinal interface, is a common disease mainly associated with aging and posterior vitreous detachment. This is mostly due to the proliferation of retinal elements, including different types of cells and proteins. The major cellular components of iERM are retinal glial cells, which are derived from Müller cells [1–3]. The prevalence of iERM is 2% in individuals under 60 years of age and 12%~20% in those over 70 years of age [4]. Patients with iERM may have no symptoms in the early stage, but they often complain of a reduction in visual acuity and distorted or blurred central vision when the macular structure is destroyed. However, its diagnosis is based on exclusion, which requires ruling out secondary causes, such as an operated retinal detachment or vascular or inflammatory retinopathies. Optical coherence tomography (OCT) is a useful method for discovering microscopic retinal changes and has been applied extensively to assist in the diagnosis of iERM [5].

Pars plana vitrectomy (PPV) and ERM removal are considered standard surgical interventions to treat symptomatic patients [6], and most patients have an improved visual outcome after surgery. Unfortunately, ERM recurs in approximately 10%~21% of patients, and 3% of patients with recurrence require reoperation [7, 8]. As the basal lamina of Müller cells, the internal limiting membrane (ILM) plays an important role in the recurrence of ERM. A previous study proposed that removing the ILM during iERM surgery can prevent the recurrence of ERM [7]. The use of indocyanine green (ICG) and brilliant blue G (BBG), seem to have improved the safety of ILM peeling, which is now being gradually accepted as a treatment modality [9]. However, surgery with ILM peeling may have a prognosis similar to that without ILM peeling. Moreover, the process of ILM peeling may damage the function of Müller cells and induce visual function deterioration, macular edema, or retinal hemorrhage.

Although previously published meta-analyses have compared the surgical effects between the methods of iERM removal with or without ILM peeling, they did not include enough randomized controlled trials (RCTs) and did not have consistent conclusions regarding postoperative best-corrected visual acuity (BCVA) and central macular thickness (CMT) [10, 11]. Although the controversy surrounding the surgical methods has been previously discussed, we included more studies on iERM to provide more reliable evidence.

## 2. Objectives

The main outcomes were short-term (<12 months) and long-term (≥12 months) BCVA improvements. The postoperative logarithm of minimum angle resolution (logMAR) at specific point minus the preoperative logMAR is defined as BCVA improvement. The secondary outcomes were short-term (<12 months) and long-term (≥12 months) CMT reduction and recurrence of ERM. CMT reduction means that the distance between the vitreous retinal interface and the inner border of the retinal pigment epithelium decreases. Recurrence of ERM is defined as either biomicroscopic or OCT evidence of recurrent macular pucker. We aimed to determine whether ERM removal with or without ILM peeling was more beneficial for iERM treatment.

## 3. Search methods

### 3.1 Literature search strategy

The terms used for searching were (A) “Epiretinal membrane [Mesh]”, (B) “Epiretinal membrane [Title and Abstract]”, (C) “Epiretinal membranes [Title and Abstract]”, (D) “Membrane, epiretinal [Title and Abstract]”, (E) “Membranes, epiretinal [Title and Abstract]”, (F) “Epimacular membrane [Title and Abstract]”, (G) “Macular pucker [Title and Abstract]”, (H) “Internal limiting membrane [Title and Abstract]”, and (I) “Inner limiting membrane [Title and Abstract]”. The search was conducted using “A or B or C or D or E or F or G” and “H or I” in the PubMed, Embase, Cochrane Library, Web of Science, and Open Grey databases. No language restrictions were imposed, and the studies included were from database inception to December 2019.

### 3.2 Criteria for inclusion and exclusion

All RCTs or retrospective studies on iERM, with or without ILM peeling, were included. The foveal-sparing ILM was considered the type without ILM peeling. In addition, at least one of the outcomes, such as BCVA, CMT, and recurrence of ERM, had to be reported in each study. Moreover, after intervention, the research subjects should have been followed up for at least 6 months. The exclusion criteria were as follows: non-availability of the abstract or full text; conference abstracts or papers; low-quality investigations; studies without complete data that would affect the results; and choosing the most comprehensive, longest observation from the republished research.

### 3.3 Data extraction

Two members of our team independently oversaw data extraction from all the included studies. In cases of disagreement, they would first discuss and try to reach a consensus; otherwise, they sought help from another more experienced researcher. The basic information collected included the first author’s name, publication date, and journal name. The study design, number and age of patients, and length of follow-up were also collected. Importantly, data on the outcomes of interest, such as BCVA, CMT, and recurrence of ERM, were extracted.

### 3.4 Quality and risk bias assessment

The quality of retrospective trials was assessed using the Newcastle-Ottawa Scale [12]. All scores of the retrospective studies were greater than 6 points separately. The RCTs were evaluated according to the Cochrane Collaboration tools [13]. We assessed the risk of bias according to the following principles: generation and concealment of the allocation sequence; blinding; attrition and exclusions; other generic sources of bias; biases specific to the trial design or specific to a clinical specialty. All included studies met the requirement for a meta-analysis.

### 3.5 Statistical analysis

We performed statistical analyses using the Review Manager 5.3 software. As for the final results, we compared five outcomes with or without ILM peeling, including short-term and long-term BCVA, short-term and long-term CMT, and recurrence rate of ERM. Data from the latest follow-up, before 12 months, were considered the short-term outcomes. The long-term outcomes were considered after 12 months and up to the end of the study. BCVA and CMT were continuous variables. We compared the mean differences (MDs) in changes between the groups with or without ILM peeling. As for the recurrence rate, the binary variable, we calculated risk ratios. All outcomes were given with 95% confidence intervals (CIs). The chi-squared test was used to determine the heterogeneity between the results of each study, and I^2^ was used to assess the heterogeneity quantitatively. An I^2^ value > 50% indicated moderate to high heterogeneity. For studies without statistical heterogeneity, the fixed-effects model was used for the combined analysis. For those with statistical heterogeneity, the random-effects model was applied. Moreover, we used funnel plots to test for possible publication bias and conducted sensitivity or subgroup analysis in order to assess the similarity of the results across subgroups.

## 4. Results

### 4.1 Study selection

**Fig 1:**
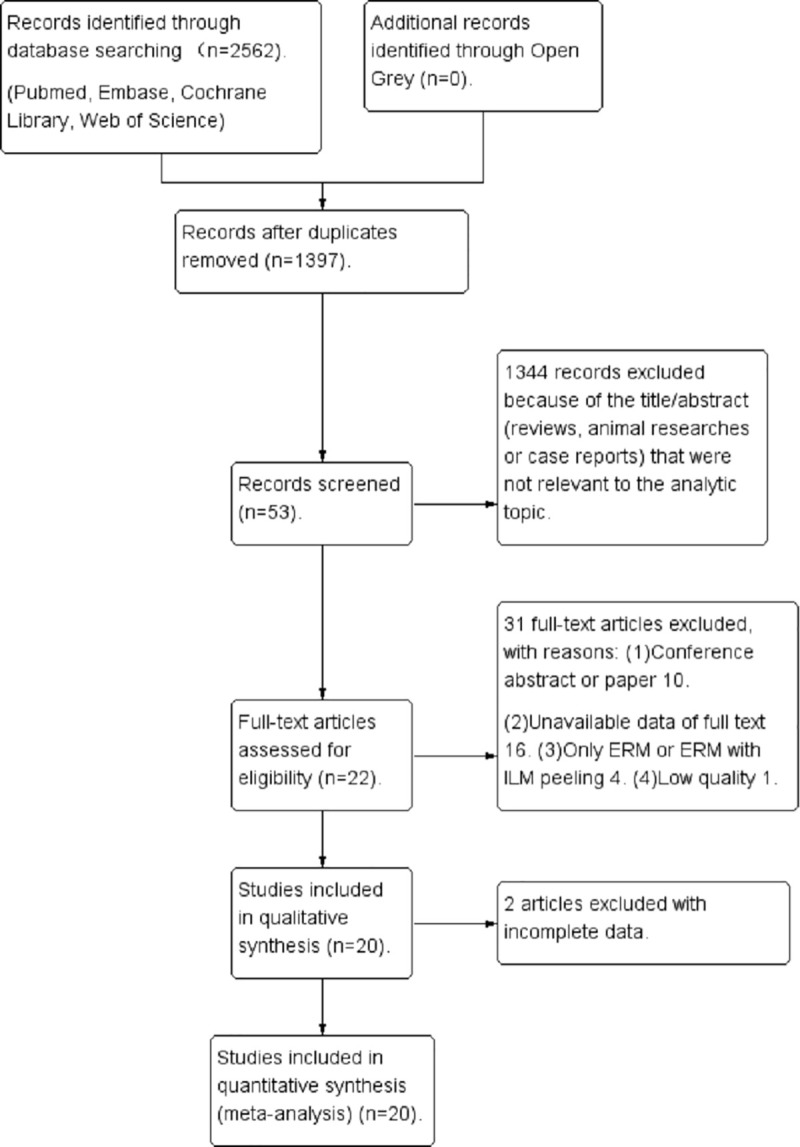
The process of identifying eligible studies.

The electronic searches of the databases initially yielded 2562 articles. Of these, 2509 articles were excluded preliminarily because these studies were reviews or duplicates, animal studies, or unrelated to our specific topic. We further excluded 33 articles from analysis: 10 were conference abstracts or papers, 16 studies were unavailable, 4 were on ERM only or ERM with ILM peeling, 1 had low quality, and 2 had incomplete data. Finally, 20 articles were used in our research, including 15 retrospective studies [6, 7, 14–26] and 5 RCTs [27–31]. The process of study selection is shown in Fig 1. The scores of the retrospective trials assessed using the Newcastle-Ottawa Scale were greater than 6 points separately [12]. Moreover, the RCTs had a low risk of bias, as evaluated using the Cochrane Collaboration tools [13].

### 4.2 Characteristics of studies

In total, 1645 eyes from 20 studies in our meta-analysis underwent treatment for iERM. The mean age of most patients was between 60 and 70 years old. The study durations ranged from 6 months to 8 years. The baseline data of preoperative BCVA and CMT are shown in Table 1. The final results of BCVA, CMT, and ERM recurrence are shown in Table 2. The quality of all studies was strictly assessed and unqualified data were excluded.

**Table 1:** The baseline characteristic of included studies (①with ILM peeling ②without ILM peeling).

Studies	Study type	Quality scores	Types	Number Of eyes at the end of study	Mean age (year)	Time of follow-up (Month)	Preoperative BCVA (Log MAR)	Preoperative CMT (μm)
Park, D. W., et al. (2003)	Retro	8	①	20	69	>12	/	/
			②	24	69	>12	/	/
Kwok, A., et al. (2005)	Retro	8	①	25	63.8+-9.3	23.9+-5.5	0.77+-0.50	/
			②	17	69.1+-8.3	47.9+-18.1	0.96+-0.18	/
Shimada, H., et al. (2009)	Retro	7	①	142	68.04	12	0.573	/
			②	104	66.17	12	0.560	/
Lee, J. W. and I. T. Kim (2010)	Retro	8	①	21	63.43+-7.18	18.08+-11.81	0.68+-0.21	409.43+-111.6
			②	19	65.47+-7.66	18.2+-12.0	0.67+-0.34	398.42+-95
Pournaras, C. J., et al. (2011)	Retro	8	①	24	73.3+-10.6	24.0+-12.6	0.58+-0.40	401+-96
			②	15	77.1+-6.7	41.9+-35.6	0.48+-0.22	/
Oh, H. N., et al. (2013)	Retro	8	①	20	65.3	12	0.44+-0.21	/
			②	23	64	12	0.35+-0.16	/
Ahn, S. J., et al. (2014)	Retro	8	①	40	64.3+-10.0	12	0.31+-0.21	445+-99.3
			②	69	63.9+-11.1	12	0.38+-0.19	456+-77.4
Kang, K. T., et al. (2014)	Retro	8	①	24	62.78 +- 10.74	709.51 +- 334.03	/	/
			②	17	62.78 +- 10.74	709.51 +- 334.03	/	/
Ripandelli, G., et al. (2015)	RCT	/	①	30	/	12	0.306+-0.214	464.20+-89.20
			②	30	/	12	0.298+-0.10	473.80+-75.70
Jung, J. J., et al. (2016)	Retro	8	①	42	71.5	29.9	0.52	0.52
			②	43	68.6	36.3	0.53	/
De Novelli, F. J., et al. (2017)	Retro	8	①	47	67.2+-1.3	36	0.60	497+-123.0
			②	78	65.8+-10.6	46	0.61	467+-85.9
Obata, S., et al. (2017)	Retro	8	①	39	69.2+-6.0	>12	0.26+-0.18	453.4+-94.6
			②	61	70.5+-7.2	>12	0.32+-0.26	463.4+-80.7
Schechet, S. A., et al. (2017)	Retro	8	①	111	67.36+-10.24	32.1	0.59+-0.03	449.79+-10.02
			②	140	68.44+-10.58	45.36	0.62+-0.02	425.40+-11.86
Tranos, P., et al. (2017)	RCT	/	①	50	68+-12	12	0.50+-0.04	512+-120
			②	52	70+-6	12	0.55+-0.05	540+-113
Lee, C. H., et al. (2018)	Retro	8	①	39	66.59+-1.41	6	0.23+-0.03	466.4+-11.31
			②	37	68.73+-1.14	6	0.27+-0.03	458.7+-10.25
Sultan, H., et al. (2018)	Retro	8	①	61	65.6+-11.6	60	0.594+-0.544	483+-121
			②	17	64.1+-10.6	60	0.948+-0.431	474+-191
Yang, X. Q. and T. Li (2018)	Retro	8	①	32	56.63+-9.8	24	0.659+-0.132	462.47+-14.287
			②	21	56.81+-10.47	24	0.676+-0.132	461.14+-13.477
De Novelli, F. J., et al. (2019)	RCT	/	①	28	67+-9.4	6	0.67+-0.29	486+-125
			①	35	66+-9.6	6	0.63+-0.31	475+-117
Russo, A., et al. (2019)	RCT	/	②	19	69.8	12	0.42+-0.11	438+-88
			①	19	72.7	12	0.43+-0.16	430+-80
Storch, M. W., et al. (2019)	RCT	/	②	5	71.7	96	0.40	390.6
			①	6	71.7	96	0.57	498.7

**Table 2:** Each outcome of included studies (①with ILM peeling ②without ILM peeling; short-term <12months, long-term ≥12months).

Studies	Types	Short-term BCVA (LogMAR)	Long-term BCVA (LogMAR)	BCVA Improvement (LogMAR) (-short-term,—long-term)	Short-term CMT (μm)	Long-term CMT (μm)	CMT reduction (μm) (-short-term,—long-term)	Recurrent rate of ERM
Park, D. W., et al. (2003)	①	/	/	--0.41	/	/	/	0/20
	②	/	/	--0.33	/	/	/	5/24
Kwok, A., et al. (2005)	①	/	0.46+-0.37	--0.31+-0.45	/	/	/	0/25
	②	/	0.65+-0.32	--0.31+-0.29	/	/	/	3/17
Shimada, H., et al. (2009)	①	/	0.266	--0.31+-0.31	/	/	/	0/142
	②	/	0.300	--0.26+-0.33	/	/	/	17/104
Lee, J. W. and I. T. Kim (2010)	①	/	0.20+-0.17	--0.48+-0.16	/	335.24+-76.91	--74.19+-79.33	0/21
	②	/	0.32+-0.23	--0.36+-0.30	/	282.53+-95.71	--115.89+-107.48	0/19
Pournaras, C. J., et al. (2011)	①	/	0.32+-0.39	--0.26+-0.4	/	307+-49	--94+-83.13	/
	②	/	0.37+-0.42	--0.09+-0.36	/	268+-98	/	/
Oh, H. N., et al. (2013)	①	0.46+-0.26	0.54+-0.26	-0.02+-0.24, --0.09+-0.25	303.8+-63.7	294.9+-64.8	-179.9+90.6, --188.8+-90.7	0/20
	②	0.43+-0.24	0.50+-0.28	-0.09+-0.21, --0.16+-0.24	293.2+-56.2	285.4+-51.4	-167.8+86.6, --175.6+-86.3	0/23
Ahn, S. J., et al. (2014)	①	0.30+-0.21	0.17+-0.17	-0.08+-0.2, --0.21+-0.18	/	342+-38.9	--104+-67	3/40
	②	0.17+-0.17	0.11+-0.12	-0.14+-0.19, --0.2+-0.18	/	356+-58.9	--89+-86.5	14/69
Kang, K. T., et al. (2014)	①	/	/	/	/	/	/	1/24
	②	/	/	/	/	/	/	1/17
Ripandelli, G., et al. (2015)	①	-0.07+-0.08	-0.05+-0.08	-0.23+-0.19, --0.26+-0.19	386.03+-47.62	376.90+-45.12	-78.17+-77.31, --87.3+-77.25	/
	②	-0.08+-0.12	-0.03+-0.11	-0.22+-0.17, --0.26+-0.17	359.03+-48.24	351.03+-40.24	-114.77+-88.38, --122.77+-65.6	/
Jung, J. J., et al. (2016)	①	0.27	0.23	-0.25,-- 0.29	/	/	--84.1+-90.2	0/42
	②	0.35	0.32	-0.18,-- 0.21	/	/	--136.9+-110.5	9/43
De Novelli, F. J., et al. (2017)	①	/	0.20	--0.4	/	367+-75.2	--130+-107.4	6/47
	②	/	0.20	--0.41	/	361+-101.1	--106+-94.4	16/78
Obata, S., et al. (2017)	①	0.06+-0.20	0.05+-0.23	-0.20+-0.19, --0.21+-0.21	384.8+-64.1	380.2+-70.0	-68.8+-83.6,--73.2+-85	8/39
	②	0.06+-0.17	0.05+-0.18	-0.26+-0.23, --0.27+-0.23	384.8+-64.1	380.2+-70.0	-78.6+-73.8, --83.2+-75.9	26/61
Schechet, S. A., et al. (2017)	①	0.54	0.37	-0.05,-- 0.22	362.75	367.22	-87.04,--82.57	2/111
	②	0.59	0.39	-0.03,-- 0.23	366.2	315.45	-59.2, --109.95	32/140
Tranos, P., et al. (2017)	①	0.23	0.20	-0.27+-0.24, --0.30+-0.24	/	/	-112+-93, --125+-103	0/50
	②	0.27	0.24	-0.28+-0.29, --0.31+-0.23	/	/	-136+-85, --134+-93	0/52
Lee, C. H., et al. (2018)	①	0.11+-0.02	/	-0.11+-0.02	378.9+-5.89	/	-87.51+-9.87	/
	②	0.16+-0.02	/	-0.11+-0.03	360.8+-8.94	/	-97.95+-8.35	/
Sultan, H., et al. (2018)	①	/	0.397	--0.197	/	/	/	18/61
	②	/	0.477	--0.471	/	/	/	8/17
Yang, X. Q. and T. Li (2018)	①	0.578+-0.101	0.511+-0.081	-0.081+-0.119, --0.148+-0.115	373.44+-8.328	274.28+-8.340	-89.03+-12.429, --188.19+-12.873	0/32
	②	0.551+-0.085	0.506+-0.032	-0.125+-0.119, --0.17+-0.124	368.52+-13.216	273.29+-8.973	-92.62+-13.348, --187.85+-11.883	4/21
De Novelli, F. J., et al. (2019)	①	0.43+-0.43	/	-0.2+-0.38	388+-69.2	/	-87+-102	1/28
	②	0.27+-0.25	/	-0.4+-0.27	377+-82.5	/	-109+-110	6/35
Russo, A., et al. (2019)	①	0.16+-0.08	0.17+-0.08	-0.26+-0.10, --0.25+-0.10	357+-36	339+-50	-81+-77, --99+-77	0/19
	②	0.16+-009	0.17+-0.06	-0.27+-0.14, --0.26+-0.14	350+-74	341+-61	-80+-77, --89+-72	3/19
Storch, M. W., et al. (2019)	①	0.38	0.10	-0.09,--0.3	347	278	-88,--113	0/5
	②	0.38	0.31	-0.16,--0.26	321	235	-147,--264	1/6

### 4.3 Outcomes

#### 4.3.1 BCVA improvement

**Fig 2:**
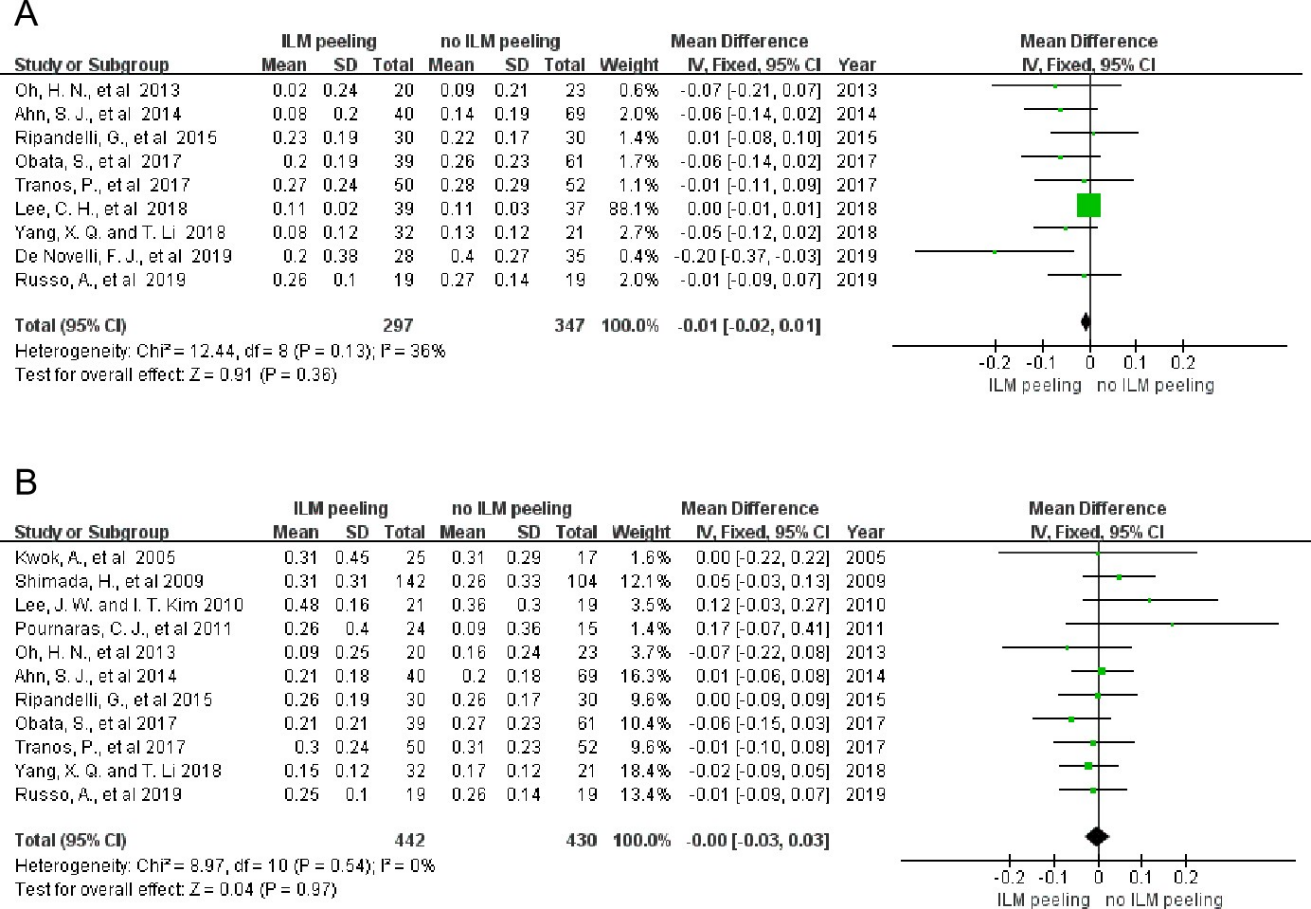
Meta-analysis comparing short-term (<12 months) (A) and long-term (≥12 months) (B) BCVA improvement between the groups with and without ILM peeling. BCVA is given as the logMAR.

**Fig 3:**
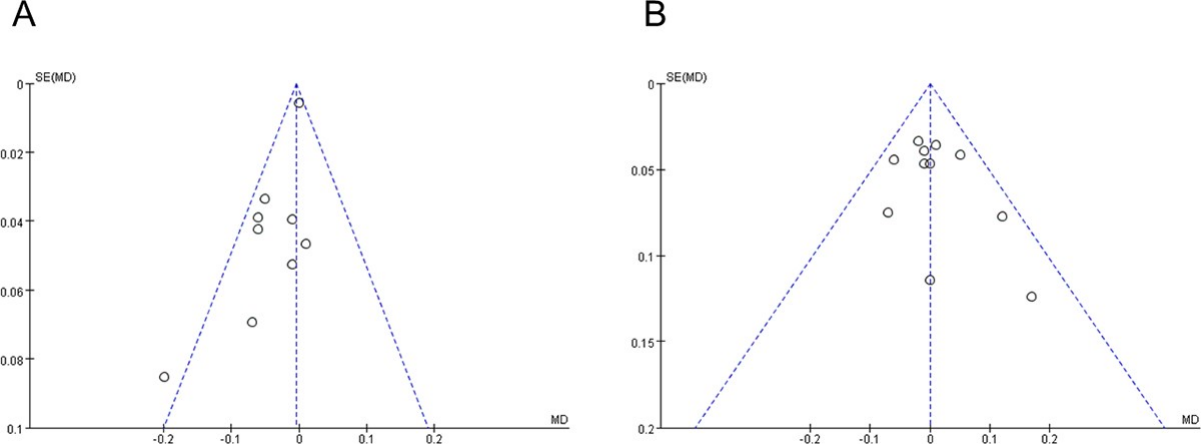
The funnel plot of short-term (<12 months) (A) and long-term (≥12 months) (B) BCVA improvement between the groups with and without ILM peeling.

The meta-analysis included 644 eyes from 9 studies on short-term BCVA improvement. No statistically significant differences were observed in heterogeneity between the two groups with or without ILM peeling (P = 0.13; I^2^ = 36%). BCVA improvement in both groups showed no significant difference (MD = -0.01; 95% CI = -0.02 to 0.01; P = 0.36) (Fig 2A). We conducted a sensitivity analysis restricting the analysis to the retrospective studies and the RCTs separately. In retrospective studies, we found that short term BCVA improvement showed no significant difference when changing the analysis model (fixed model: P = 0.13, I^2^ = 43%, MD = -0.00, 95% CI = -0.01 to 0.01, P = 0.46; random model: P = 0.13, I^2^ = 43%, MD = -0.03, 95% CI = -0.07 to 0.01, P = 0.09). In RCTs, we also found that short term BCVA improvement showed no significant difference when changing the analysis model (fixed model: P = 0.17, I^2^ = 40%, MD = -0.02, 95% CI = -0.07 to 0.03, P = 0.41; random model: P = 0.17, I^2^ = 40%, MD = -0.03, 95% CI = -0.09 to 0.04, P = 0.40). It reveals that the result of short term BCVA improvement in our meta-analysis is steady and credible. The funnel plot hinted at a potential bias in the results (Fig 3A). Once the article of Lee et al. [24] was excluded, BCVA improvement was significantly better in the eyes with ILM peeling than in those without ILM peeling (MD = -0.04; 95% CI = -0.07 to -0.01; P = 0.008). However, we found that there was not obvious bias when we analyzed the bias including article of Lee et al. with Egger’s regression (P = 0.945 >0.05). Thus, we included the article of Lee et al. in our analysis of short-term BCVA improvement.

The heterogeneity in long-term BCVA improvement in 872 eyes from 11 studies with and without ILM peeling was not statistically significant (P = 0.54; I^2^ = 0%). The fixed-effects model showed no statistical difference in the results of BCVA improvement (MD = -0.00; 95% CI = -0.03 to 0.03; P = 0.97) (Fig 2B). There was also potential bias in the results showed by funnel plot because of a limited number of included studies. (Fig 3B).

#### 4.3.2 CMT reduction

**Fig 4:**
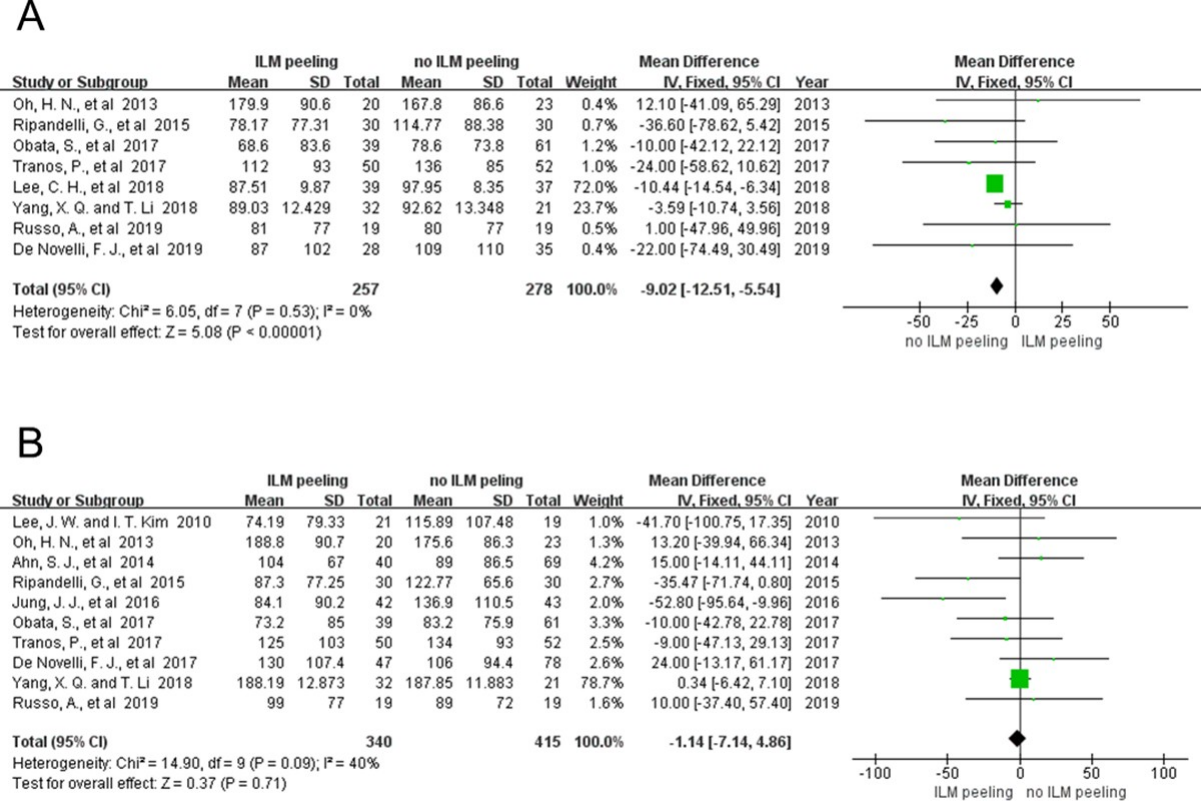
Meta-analysis comparing short-term (<12 months) (A) and long-term (≥12 months) (B) CMT reduction between the groups with and without ILM peeling.

**Fig 5:**
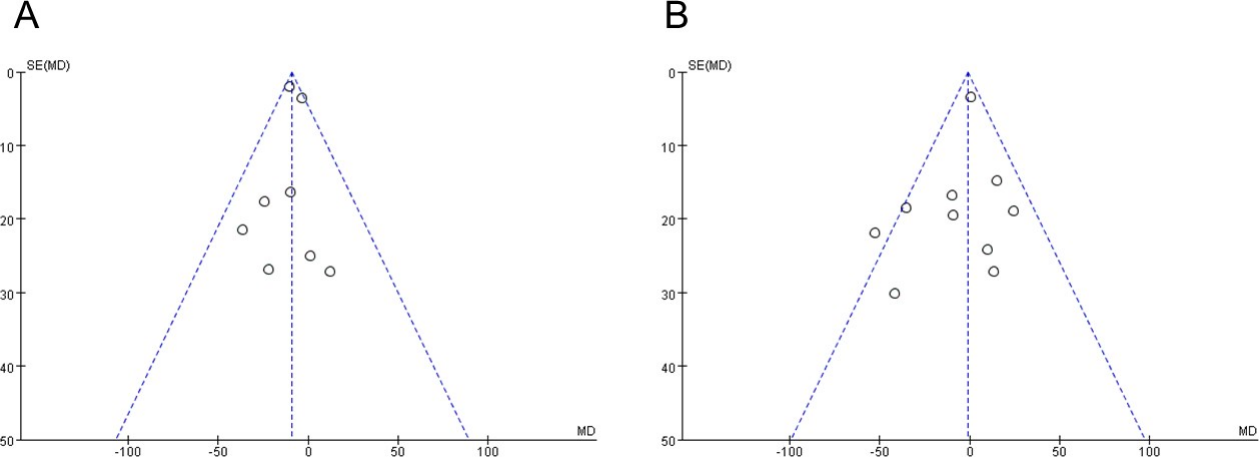
The funnel plot of short-term (<12 months) (A) and long-term (≥12 months) (B) CMT reduction between the groups with and without ILM peeling.

We compared the short-term decrease in CMT in 535 eyes from 8 studies with and without ILM peeling. The data showed that the decrease in CMT was statistically significant between the studies with and without ILM peeling (MD = -9.02; 95% CI = -12.51 to -5.54; P < 0.00001), with no significant heterogeneity (P = 0.53; I^2^ = 0%) (Fig 4A). There was potential bias in the results showed by funnel plot because of limited included studies. (Fig 5A).

We also compared the decrease in long-term CMT in 755 eyes from 10 studies. The results showed mild heterogeneity (P = 0.09; I^2^ = 40%). However, no statistically significant decrease in long-term CMT was observed between the groups with and without ILM peeling (MD = -1.14; 95% CI = -7.14 to -4.86; P = 0.71) (Fig 4B). In the subgroup analysis, the heterogeneity decreased and the significance of the total effect was the same before and after excluding the study by Jung et al. [20] (after exclusion: P = 0.33; I^2^ = 13%; MD = -0.10; 95% CI = -6.16 to -5.96; P = 0.97). Potential bias could not be ignored because the included studies were limited (Fig 5B).

#### 4.3.3 Recurrence of ERM

**Fig 6:**
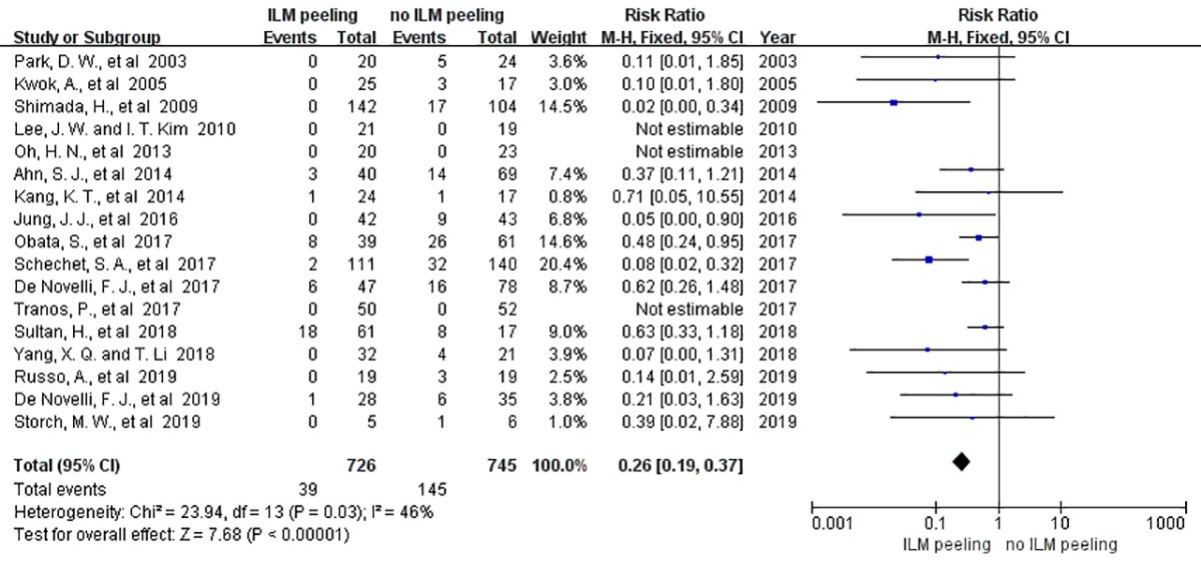
Meta-analysis comparing the recurrence of ERM between the groups with and without ILM peeling.

**Fig 7:**
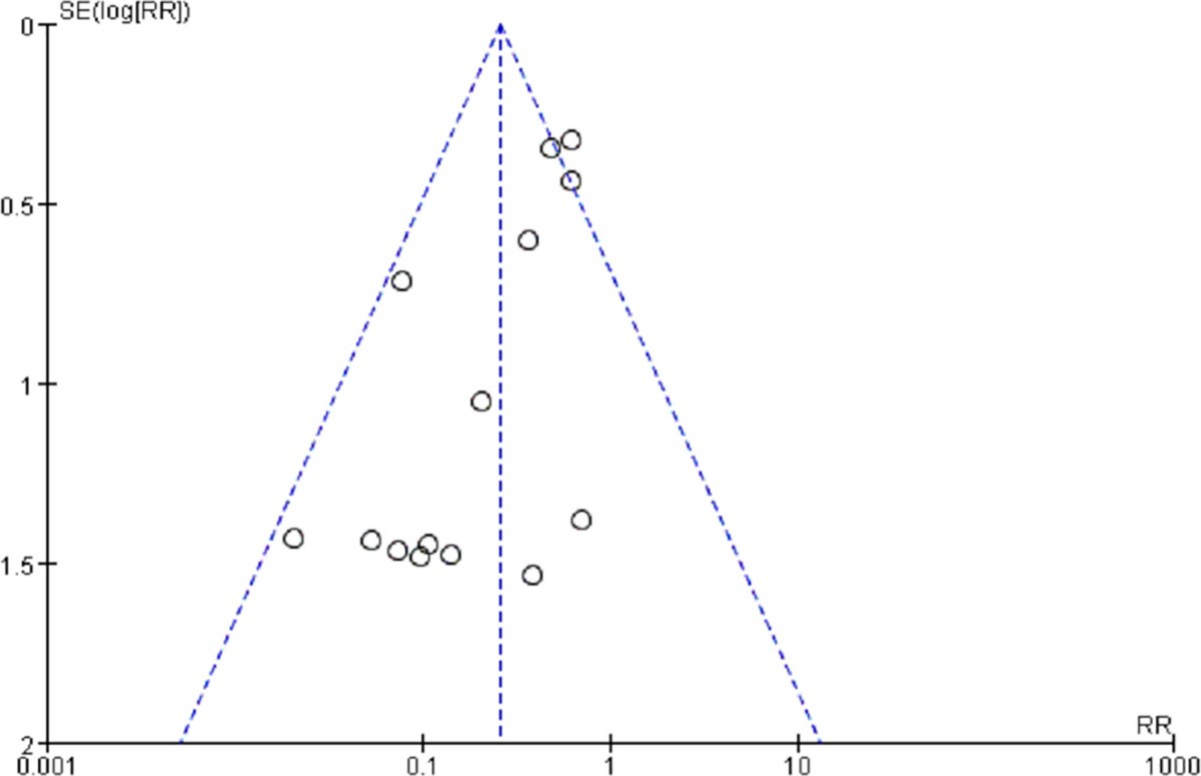
The funnel plot of recurrence of ERM between the groups with and without ILM peeling.

Recurrence of ERM was analyzed in 1471 eyes from 17 studies. Mild heterogeneity was observed between these studies (P = 0.03; I^2^ = 46%; RR = 0.26; 95% CI = 0.19 to 0.37). This showed that the recurrence of ERM in groups with or without ILM peeling was statistically significant (P < 0.00001) (Fig 6). In the sensitivity analysis, we changed the fixed model to random model and we found that the result did not change obviously (P = 003; I^2^ = 46%; RR = 0.28; 95% CI = 0.16 to 0.49). Moreover, the recurrence of ERM in groups was also statistically significant (P < 0.00001). It means that the result of recurrence of ERM in our meta-analysis is steady and credible. The funnel plot showed no publication bias (Fig 7). [23] l [25].

## 5. Discussion

In our meta-analysis, we analyzed BCVA, CMT, and recurrence of ERM in 20 studies. The data which was lacking in standard deviation was excluded in analysis of the above items. As a result, there were some studies that could not be included. Although the risk of bias in RCTs was at a low level totally, there was some unclear and high risk of bias in some studies indeed. The prognosis in terms of short-term BCVA was the same between the groups with and without ILM peeling. After exclusion of an original study [24], the result changed and short-term BCVA was better in the group with ILM peeling than in that without ILM peeling. However, there was not obvious bias when we analyzed the bias including article of Lee et al. with Egger’s regression. Thus, we included the article of Lee et al. in our analysis of short-term BCVA improvement. Short-term CMT in the group without ILM peeling decreased more obviously than it did in the group with ILM peeling, and the recurrence of ERM was lower in the group with ILM peeling than in that without ILM peeling. However, long-term BCVA and long-term CMT improved similarly in the groups with and without ILM peeling.

Another meta-analysis published several years ago did not reach an agreement on the treatment for iERM. Chang [10] analyzed 12 studies involving 756 eyes and found that PPV with ILM peeling had better outcomes in terms of long-term (>18 months) BCVA and lower ERM recurrence rate. The reduction in CMT was more apparent in the group without ILM peeling. Nevertheless, Kumihiro Azuma [11] arrived at another conclusion by analyzing 15 studies involving 1367 eyes. Compared with the absence of ILM peeling, additional ILM peeling did not significantly affect BCVA and CMT after the intervention, but it could reduce the recurrence of ERM. As a result, both methods were used by different surgeons according to their personal experience. We aimed to provide more reliable evidence supporting iERM treatment.

The visual acuity of patients with iERM will reduce when the inner retinal layers contort. Retinal thickening and edema are the main causes of distortion. However, the optimal time of surgical intervention is difficult to determine. On the basis of morphological characteristics evaluated using spectral-domain OCT (SD-OCT), iERM was classified into five groups. The macular function of each group can be demonstrated by applying multifocal electroretinography, which is an objective and noninvasive method that helps detect regional functional changes in the central retina by measuring electrophysiological responses. It can be used to assess the degree of functional decline in the foveal area and provide evidence supporting surgery [32, 33].

Initially, ERM peeling was the major surgical method for symptomatic patients with iERM. However, studies analyzing the histological features of surgical specimens discovered that the ILM was removed spontaneously together with ERM peeling in some cases, and reported that iERM was less likely to have focal points of adhesion to the retina than does secondary ERM [34, 35]. Meanwhile, some patients experienced recurrence of ERM after treatment, and some of them needed further surgery. Incomplete iERM removal is believed to result in the regrowth of myofibroblasts and to lead to the recurrence of ERM [36]. The ILM provides a scaffold for proliferation, and hence the method of iERM treatment with ILM peeling was proposed [15]. However, peeling the ILM is difficult because of its transparent nature. ICG was first used during the process of surgery, and it provided a clear distinction between the stained ILM and unstained retina [37]. Moreover, it could protect the retina from mechanical injury during ILM peeling and increase the surgeons’ confidence in performing the surgery. Later, the safety of ICG was discussed, and it was discovered that its concentration, osmolarity, and time of tissue contact could cause potential damage to the retina [38]. Choi, W. S., et al. [39] also concluded that intravitreal ICG-assisted ILM peeling did not influence the recovery of BCVA but impaired the recovery of CMT. In another study, BBG was considered the state-of-the-art dye for ILM identification and was gradually used to assist in ILM peeling [40]. Recently, intraoperative OCT-guided ERM peeling has demonstrated similar visual outcomes and anatomic improvements without ILM peeling, when compared with the conventional method [41]. Although the methods of ILM peeling have been updated, analysis of risk bias reveals that short-term BCVA does not seem to improve in the groups with or without ILM peeling in our meta-analysis. The result of our meta-analysis also revealed a similar level of improvement in long-term BCVA in both groups. We speculate that iERM is the main factor affecting the macular structure and that BCVA improves after iERM removal.

CMT decreased significantly following iERM removal with or without ILM peeling [42]. Oh, H. N., et al. [17] and Sultan, H., et al. [25] reported that the short-term decrease in CMT was higher in the group with ILM peeling than in that without ILM peeling. On the contrary, most studies revealed a more severe decrease in CMT in the group without ILM peeling. The development of modalities such as SD-OCT improved the evaluation of iERM and have allowed accurate measurement of changes in CMT [43]. The short-term changes in CMT between the two groups may be due to the different levels of decrease in tissue structure; moreover, the degree of reactive edema may influence the results. Ocular reactions can reduce over time, and the long-term changes in CMT subsequently seem similar. In addition, we found that almost all included studies showed a lower recurrence of ERM in the group with ILM peeling. As mentioned previously, ILM peeling not only removes the residual ERM, but also eliminates the scaffold for cell proliferation. It is not controversial that the ILM peeling is beneficial for reduction of ERM recurrence.

Although ILM peeling does not seem to have an adverse effect on BCVA and CMT, it is not totally without side effects. When peeling the ILM, mechanical injury to the retina may still occur. The histological disorganization may also lead to microscotomas and result in visual discomfort or even macular edema. Moreover, the mean retinal sensitivity showed a slow recovery in patients with ILM peeling [27, 44].

Our meta-analysis included more original studies than previous meta-analyses. However, the number of studies analyzing each outcome seems insufficient. Moreover, heterogeneity exists because of the combination of different types of studies. Furthermore, we did not impose strict restrictions on factors such as age, cataract surgery, and follow-up time, and these may have influenced the results. Last but not least, the risk of bias in included studies could not be ignored. The bias, such as selection bias, performance bias, detection bias and so on, might influence the final result of meta-analysis. We suggest that higher-quality RCTs be included in future meta-analyses to obtain more reliable results.

## 6. Conclusion

By considering the risk of bias, we should determine whether ILM peeling is beneficial for short-term changes in BCVA in patients with iERM. Nevertheless, further studies are needed to confirm this. iERM removal without ILM peeling can improve the short-term decrease in CMT and ILM peeling decreases the recurrence of ERM, but the long-term changes in BCVA and CMT are similar with or without ILM peeling. There is a need for a true large scale randomized trial that will also include microperimetry and other functional measures.

## Supporting information

S1 Checklist PRISMA 2009 checklist. (DOC)

S1 Fig The funnel plot after having excluded the study of Lee et al. in analysis of short-term BCVA improvement. (DOCX)

S2 Fig The result of Egger’s regression test in analysis of short-term BCVA improvement. (DOC)

S1 Table The publication is not included because of the unavailable abstract or full text. (DOCX)

S2 Table The publication is not included because of incomplete data. (DOCX)

S3 Table Risk of bias summary in RCTs. (DOCX)

S1 Flow diagram PRISMA 2009 flow diagram. (DOC)
