# Supplementary material for: With or without internal limiting membrane peeling during idiopathic epiretinal membrane surgery: A meta-analysis
Source: PLoS One. 2021 Jan 19;16(1):e0245459. doi: 10.1371/journal.pone.0245459 (PMC7815136; doi:10.1371/journal.pone.0245459)

S1 Fig: The funnel plot after having excluded the study of Lee et al. in analysis of short-term BCVA improvement.


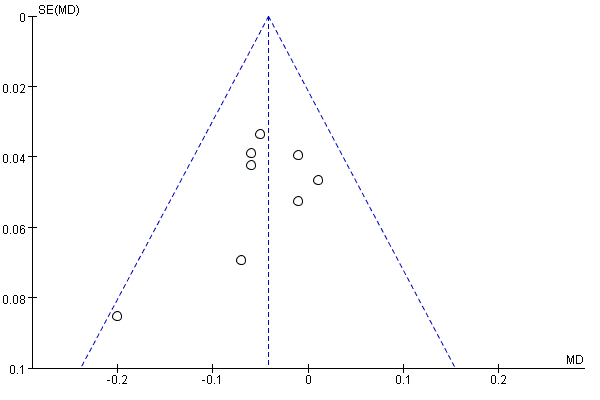

Supplement: S1 Fig — (DOCX) [file pone.0245459.s002.docx]
