# Supplementary figures and images for: With or without internal limiting membrane peeling during idiopathic epiretinal membrane surgery: A meta-analysis
Source: PLoS One. 2021 Jan 19;16(1):e0245459. doi: 10.1371/journal.pone.0245459 (PMC7815136; doi:10.1371/journal.pone.0245459)

S2 Fig: The result of Egger’s regression test in analysis of short-term BCVA improvement.


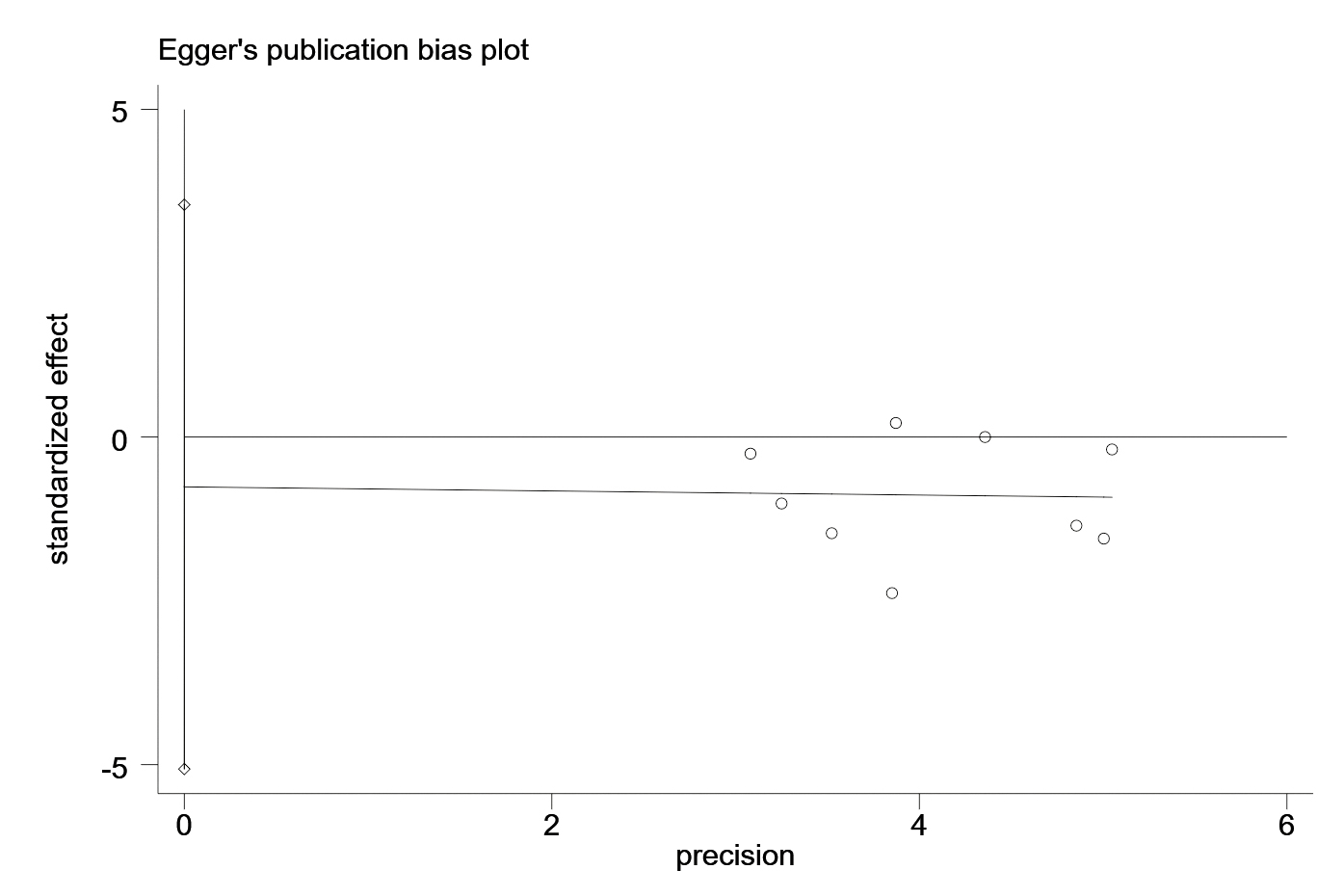

Supplement: S2 Fig — (DOC) [file pone.0245459.s003.doc]

S3 Table: Risk of bias summary in RCTs.


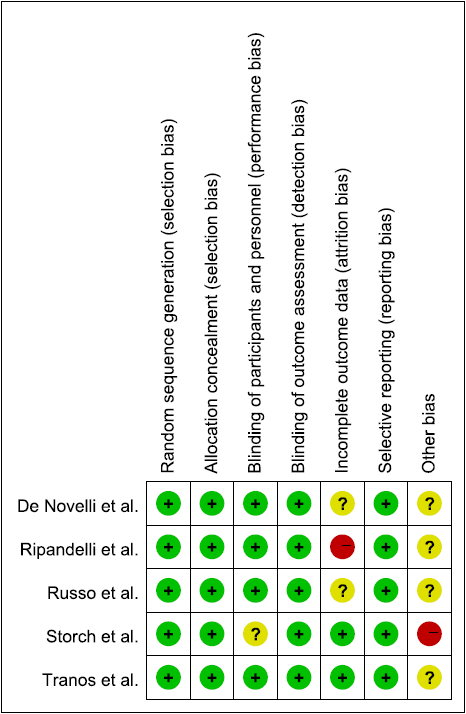


▇ Low risk of bias ▇ Unclear risk of bias ▇ High risk of bias

Supplement: S3 Table — (DOCX) [file pone.0245459.s006.docx]
