# Supplementary material for: With or without internal limiting membrane peeling during idiopathic epiretinal membrane surgery: A meta-analysis
Source: PLoS One. 2021 Jan 19;16(1):e0245459. doi: 10.1371/journal.pone.0245459 (PMC7815136; doi:10.1371/journal.pone.0245459)
Supplement: S1 Table — (DOCX) [file pone.0245459.s004.docx]

S1 Table: The publication is not included because of the unavailable abstract or full text.

| No. | Title | Authors and Publication time |
| --- | --- | --- |
| 1 | Ablation of the internal limiting membrane, macular unfolding and visual outcome in surgery of idiopathic epimacular membranes | Gaudric A et al., 1993 |
| 2 | Foveal thickness and retinal sensitivity following surgery for idiopathic epiretinal membrane | Kumagai et al., 2000 |
| 3 | Evaluation of peeling of inner limiting membrane for idiopathic epiretinal membrane | Liu Y et al., 2001 |
| 4 | Internal limiting membrane peeling in surgical treatment of macular epiretinal membrane | Kim TW et al., 2005 |
| 5 | Epiretinal Membrane Surgery: with and Without Internal Limiting Membrane Removal | Oncel M, 2005 |
| 6 | Outcome of vitrectomy for epimacular membrane | Nakamura H et al., 2005 |
| 7 | Visual outcomes and recurrence rates of macular pucker surgery with and without internal limiting membrane peeling | Mason IJO et al., 2006 |
| 8 | Randomized trial comparing epiretinal membrane surgery with and without internal limiting membrane removal | Nct, 2012 |
| 9 | Prospective, Single-blind Study Assessing the Benefit/Risk Ratio of Internal Limiting Membrane (ILM) Peeling During Epimacular Membrane (EMM) Surgery (Peeling) | Nct, 2014 |
| 10 | Internal limiting membrane peeling in epiretinal membrane surgery, is it a safe procedure | ÇAkir A et al., 2017 |
| 11 | Necessity of internal limiting membrane peeling in treatment of epiretinal membrane | Jprn U, 2018 |
| 12 | Effects of combined with or without internal limiting membrane peeling on macular structure and function during idiopathic epiretinal membrane surgery: a randomized controlled trial | ChiCtr, 2019 |
| 13 | Effects of internal limiting membrane peeling on visual function in epiretinal membrane surgery | Nct, 2019 |
