# Supplementary material for: With or without internal limiting membrane peeling during idiopathic epiretinal membrane surgery: A meta-analysis
Source: PLoS One. 2021 Jan 19;16(1):e0245459. doi: 10.1371/journal.pone.0245459 (PMC7815136; doi:10.1371/journal.pone.0245459)
Supplement: S2 Table — (DOCX) [file pone.0245459.s005.docx]

S2 Table: The publication is not included because of incomplete data.

| No. | Title | Authors | The reasons |
| --- | --- | --- | --- |
| 1 | Surgery for epimacular membrane: impact of retinal internal limiting membrane removal on functional outcome | Bovey EH et al., 2004 | The visual acuity does not include standard deviation and other outcomes are lack. |
| 2 | Double peeling during vitrectomy for macular pucker: the Charles L. Schepens Lecture | Chang S et al., 2013 | The standard deviation is not included in visual acuity and central macular thickness. |
| 3 | Epiretinal membrane recurrence: incidence, characteristics, evolution, and preventive and risk factors | Sandali O et al., 2013 | The included patients are with primary or secondary epiretinal membrane. There is not specific data for patients with primary epiretinal membrane. |
| 4 | Outcomes in patients with macular pucker and good preoperative visual acuity after vitrectomy with membrane peeling | Reilly G et al., 2015 | The visual acuity does not include standard deviation and other outcomes are lack. |
| 5 | Outcomes after Epiretinal Membrane Surgery with or Without Internal Limiting Membrane Peeling | Guber J et al., 2019 | The standard deviation is not included in visual acuity and central macular thickness. |
