# Supplementary material for: With or without internal limiting membrane peeling during idiopathic epiretinal membrane surgery: A meta-analysis
Source: PLoS One. 2021 Jan 19;16(1):e0245459. doi: 10.1371/journal.pone.0245459 (PMC7815136; doi:10.1371/journal.pone.0245459)
Supplement: S1 Flow diagram — (DOC) [file pone.0245459.s007.doc]

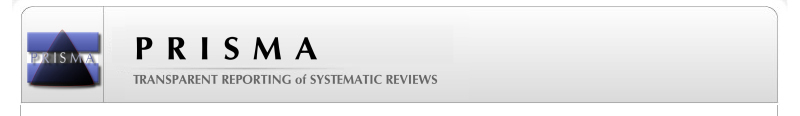
**PRISMA 2009 Flow Diagram**

**Screening**

**Included**

**Eligibility**

**Identification**

Records identified through database searching
(n = 2562 )

Additional records identified through other sources
(n = 0)

Records after duplicates removed
(n = 1397 )

Records screened
(n = 53 )

Records excluded
(n = 1344 )

Full-text articles assessed for eligibility
(n = 22)

Full-text articles excluded, with reasons
(n = 31 )

Studies included in qualitative synthesis
(n = 20 )

Studies included in quantitative synthesis (meta-analysis)
(n = 20 )
